# A bioinformatics approach for identifying transgene insertion sites using whole genome sequencing data

**DOI:** 10.1186/s12896-017-0386-x

**Published:** 2017-08-15

**Authors:** Doori Park, Su-Hyun Park, Yong Wook Ban, Youn Shic Kim, Kyoung-Cheul Park, Nam-Soo Kim, Ju-Kon Kim, Ik-Young Choi

**Affiliations:** 1 Department of Agriculture and Life Industry, Kangwon National University, 1 Kangwondaehak-gil, Chuncheon, Gangwon 24341 Republic of Korea; 2 Graduate School of International Agricultural Technology and Crop Biotech Institute/GreenBio Science and Technology, Seoul National University, 1447, Pyeongchang, Gangwon, 25354 Republic of Korea; 3 Department of Molecular Bioscience, Kangwon National University, 1 Kangwondaehak-gil, Chuncheon, Gangwon 24341 Republic of Korea; 4 Department of Forest Resources, Kangwon National University, 1 Kangwondaehak-gil, Chuncheon, Gangwon 24341 Republic of Korea; 5 Bioherb Research Institute, Kangwon National University, 1 Kangwondaehak-gil, Chuncheon, Gangwon 24341 Republic of Korea; 6 Present address: Laboratory of Plant Molecular Biology, The Rockefeller University, 1230 York Avenue, New York, NY 10065 USA

**Keywords:** Genetically modified organism (GMO), GM rice, Next-generation sequencing (NGS), Molecular characterization, GM safety, Bioinformatics

## Abstract

**Background:**

Genetically modified crops (GM crops) have been developed to improve the agricultural traits of modern crop cultivars. Safety assessments of GM crops are of paramount importance in research at developmental stages and before releasing transgenic plants into the marketplace. Sequencing technology is developing rapidly, with higher output and labor efficiencies, and will eventually replace existing methods for the molecular characterization of genetically modified organisms.

**Methods:**

To detect the transgenic insertion locations in the three GM rice gnomes, Illumina sequencing reads are mapped and classified to the rice genome and plasmid sequence. The both mapped reads are classified to characterize the junction site between plant and transgene sequence by sequence alignment.

**Results:**

Herein, we present a next generation sequencing (NGS)-based molecular characterization method, using transgenic rice plants SNU-Bt9–5, SNU-Bt9–30, and SNU-Bt9–109. Specifically, using bioinformatics tools, we detected the precise insertion locations and copy numbers of transfer DNA, genetic rearrangements, and the absence of backbone sequences, which were equivalent to results obtained from Southern blot analyses.

**Conclusion:**

NGS methods have been suggested as an effective means of characterizing and detecting transgenic insertion locations in genomes. Our results demonstrate the use of a combination of NGS technology and bioinformatics approaches that offers cost- and time-effective methods for assessing the safety of transgenic plants.

**Electronic supplementary material:**

The online version of this article (doi:10.1186/s12896-017-0386-x) contains supplementary material, which is available to authorized users.

## Background

Domesticated plants and animals have been modified (using artificial selection and crossbreeding) to meet human needs for at least 10,000 years. However, these domestication processes have resulted in severe genetic erosion in modern crops and animals, which has left them vulnerable to biotic and abiotic stresses [1]. In response, many genetic modification techniques have been invented to improve modern crop cultivars and domesticated animals [2]. One such method of creating genetically modified (GM) crops involves introducing random insertions of recombinant DNA into genomes of crops or animals through transformation techniques, such as *Agrobacterium*-mediated transformation and particle bombardment [3, 4]. However, several issues related to the production of genetically modified (GM) crops are still under debate. These issues include potential ecological impacts, concerns for food safety, and the genetic stability of crops [5, 6].

Before releasing new GM crop varieties into the marketplace, molecular characterization of the modified plants is a critical step for assessing their safety and obtaining regulatory approvals [7]. Weber et al. [8] discussed the potential risks of genomic instability on the genomic plasticity of GM plants and its effects on the safety of food and animal feed [8]. They concluded that there is no evidence that GM plants are less stable than non-GM plants and that the risks of introducing new food hazards into the food supply are no different than introducing food derived from conventional breeding process. Even so, practical concerns remain regarding the genomic locations of transgenic integrations [9, 10]. If the transgene integrates into a heterochromatin region, then it will not be expressed as desired; alternatively, if the transgene is inserted by using a strong promoter, then its expression will be suppressed by host gene silencing mechanisms.

Molecular characterization is necessary for the commercialization of transgenic crops. Using a sequence-specific probe that is homologous to the transgene, Southern blot (SB) analysis has been widely used in molecular characterization of transgenic events to determine the presence, and copy numbers of, transgenes [11, 12]. Transgenes can also be detected through polymerase chain reaction (PCR) and Sanger sequencing [13, 14] or using microarray methods [15]. Although SB and other methods are typically carried out for the selection of GM events, these methods are laborious and time consuming and suffer from limitations such as detection of single base substitutions or small insertions/deletions, which can occur within the transfer DNA (T-DNA) and its insertion site [16]. Recently, several publications showed sufficient evidence that next generation sequencing (NGS) could be used to replace or complement traditional methods [17–20]. NGS technologies will likely offer rapid and cost-effective protocols for detecting the exact copy numbers and genomic locations of transgenes, the presence of vector backbones, and the stability of T-DNA across generations. NGS is also sensitive enough to identify nucleotide substitutions other than SNPs, including small insertions and deletions, which enable comparative studies across events and reference genomes [16].

In this paper, three T_3_ GM rice plants, (*Oryza sativa japonica* cv. Illmi) SNU-Bt9–5, SNU-Bt9–30, and SNU-Bt9–109, were characterized by NGS technique before commercialization. Transfer DNA carried an insect-resistance gene, *Cry1Ac*, and plants were transformed using *Agrobacterium*-mediated transformation. From high throughput re-sequencing and bioinformatics analysis, we identified the precise genomic locations of the transgene insertions and determined the presence or absence of plasmid backbone sequences in GM plants. We intended to provide insights into the distinct advantages of applying various bioinformatics approaches, particularly regarding the molecular characterization of transgenic insertions and the application of NGS to understanding the molecular nature of transgenic genomes.

## Methods

### Rice transformation and DNA extraction

The conventional rice variety (*Oryza sativa japonica* cv. Illmi) was used as the background cultivar to generate transgenic rice lines. T-DNA was composed of the rice *ribulose-1,5-bisphosphate carboxylase/oxygenase small subunit 3* (*RbcS3*) promoter linked to transit peptide (*TP3*), insect-resistance gene *Cry1Ac* (*Bt9*), terminator *PinII* and herbicide resistant gene expression cassette (*35S::bar::3’nos*), which was used as a selection marker (Additional file 1: FigureS1). Transgenic rice lines were obtained by *Agrobacterium*-mediated co-cultivation (detailed information available in [21]). Three T_3_ homozygous transgenic rice plants, SNU-Bt9–5, SNU-Bt9–30, and SNU-Bt9–109, were used as plant materials, and Illmi rice was used as a control. Genomic DNA from 0.5 g of leaf tissue was isolated from each of the four plants using a modified DNAzol®ES method (Molecular Research Center, Cincinnati, OH, USA).

### Sequencing library preparation and whole genome sequencing

Genomic DNA quality was evaluated by 0.5% agarose gel electrophoresis. Following a quality check, genomic DNA for shotgun sequencing was sheared to a 500 bp average fragment size (Covaris, Woburn, MA, USA). A Truseq DNA PCR-free Library Preparation Kit (Illumina Inc. San Diego, CA, USA) was used to construct DNA libraries following the manufacturer’s protocol. The quality of constructed DNA libraries was confirmed by using the LabChip GX system (PerkinElmer, Waltham, MA, USA). DNA libraries were sequenced with 150-bp paired-end sequencing on an Illumina Hiseq 2500. All reads are available in the NCBI Sequence Read Archive repository [SRX2762614, SRX2762615, SRX2762616, SRX2762617, SRX2762618, and SRX2762619].

### T-DNA insert site analysis

**Fig. 1:**
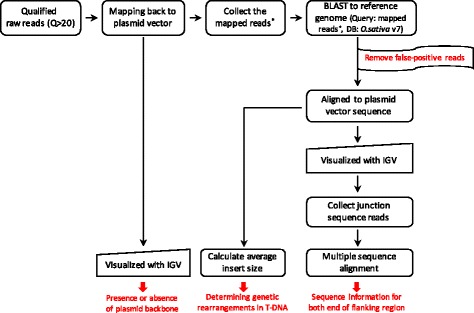
Workflow for detection of junction insertion sites

Initially, Illumina paired-end sequencing reads with average Phred scores ≥20 were retained, and duplicate sequences were removed using FastQC. These qualified reads were classified into three groups: 1) reads derived from rice endogenous genomic regions; 2) reads derived from a plasmid sequence containing transfer DNA; and 3) reads derived from the location of transgene integration sites that spanned the junction between plant and transgene sequences. To obtain the third group of reads, reads were first mapped back to transformation plasmid vector sequences (pPZP200 including T-DNA) using the Burrows-Wheeler Aligner with maximum exact matches (BWA-MEM) with a minimum seed length = 50 and band width = 2 while keeping the other default parameters [22]. Mapped reads were then used as queries against the rice reference genome (*Oryza sativa* version 7.0) using BLAST (version 2.6.0), and reads were classified as false-positive if they aligned to rice endogenous gene *rbcS3* (Os12g0291100) with an e-value of 1 × 10^−5^. The remaining reads were aligned against the entire transformation plasmid sequences and visualized in the Integrative Genomic Viewer (IGV). From the IGV results, reads that matched against both ends of the T-DNA were collected and subjected to multiple sequence alignments to identify the insert junction location on the rice chromosome. The inserted junction location was identified using NCBI-BLAST against the rice reference genome (*O. sativa*). A work-flow diagram of the search for insertion sites is given in Fig. 1, and the source code used in this study is given in Additional file 2.

## Results

### Bioinformatic workflow for the molecular characterization of GM rice events

Many researchers have difficulties in handling large quantities of bioinformatic data. We developed a user-friendly method for detecting inserted T-DNA junctions using NGS data in place of conventional detection methods. A diagram of the bioinformatic workflow is shown in Fig. 1. In the first step, qualified raw paired-end reads were aligned against a transformation plasmid vector using the Burrows-Wheeler Aligner software with maximal exact matches (BWA-MEM) [22]. As the structure of the transformation plasmid vector is circular, we made a linearized vector reference sequence (pPZP200) where both left and right border sequences contained 150 bp of the opposite end of the plasmid sequence. For selecting those reads spanning junctions, mapped reads were subtracted according to their mapped positions, based on the T-DNA location (from 6392 to 10,291 bp). These collected reads were used as queries for BLASTN analysis to classify false-positive reads against a reference rice genome (*O. sativa* version 7.0) [23]. As the inserted T-DNA is designed to contain endogenous elements, reads that contained the endogenous promoter sequence *RbcS3* were carefully removed based on sequence similarity score (to the native rice sequence) to reduce ambiguous alignment. The remaining reads were aligned against the transgenic vector and visualized using IGV with paired-end reads. From the results, we selected junction reads that partially matched both ends of the T-DNA (i.e., reads that spanned both T-DNA and the rice genome) and extracted FASTA sequences to identify the inserted T-DNA in the junction region of the genome (Fig. 1).

### T-DNA location and copy number

Approximately 28 GB of raw sequence data, corresponding to 72× sequencing depth, were obtained from the control parent cultivar “Illmi”. In addition, 30 GB, 21 GB, and 26 GB of raw data were obtained from SNU-Bt9–5, SNU-Bt9–30, and SNU-Bt9–109, respectively, representing approximately 78×, 54×, and 68× genome coverage, respectively (Table 1).

**Table 1:** Sequencing summary

Plasmid	Transgenic event	Raw data			Quality trimming (Quality 20)			Duplication removal		
		No. of reads	Read length (bp)	Coverage (X)^a^	Passing no	Passing length	%	Passing no	Passing length	%
Control	Illmi-WT	223,313,710	28,137,527,460	72	176,408,832	21,435,272,583	76	170,625,468	20,741,720,376	74
pSRT-Bt9	5	239,832,008	30,218,833,008	78	204,794,658	25,037,891,856	83	188,241,490	22,995,894,420	76
	30	166,881,634	21,027,085,884	54	138,820,046	16,967,747,284	81	133,943,766	16,376,472,378	78
	109	209,895,370	26,446,816,620	68	160,918,378	19,499,272,073	74	156,176,474	18,927,586,888	72

^a^Rice genome coverage (estimated genome size: 389 Mb [30])

From the consecutive steps applied in our junction detection analysis (as described in the ‘T-DNA insert site analysis’ section of the Methods), 11,539 reads were obtained from the GM rice SNU-Bt9–5, including 2790 paired mapped reads. Additionally, 8371 and 9767 reads were mapped from the GM rice SNU-Bt9–30 and SNU-Bt9–109, respectively, including 1792 and 2336 proper pairs of reads, respectively (Table 2). Unexpectedly, 8125 reads derived from wild-type “Illmi” were mapped to the transgenic vector sequences, including only 648 proper pairs of reads. The remaining unpaired paired-end reads were assumed to be due to a feature of Illumina sequences that can be caused by short sequence length. Also of note is that our T-DNA construct used in this study was designed to contain the rice endogenous promoter gene *rbcS3* (Os12g0291100), which takes up 1824 bp of T-DNA and is expressed on rice chromosome 12 [24]. To eliminate deceptive false-positive reads originating from the native genome (i.e., not from T-DNA), each mapped sequence was compared to the rice reference sequence using BLASTN. A total of 915, 1019, 729, and 899 reads corresponding to Illmi rice, SNU-Bt9–5, SNU-Bt9–30 and SNU-Bt9–109, respectively, all aligned to chromosome 12 and were classified as false positives.

**Table 2:** Paired-end read mapping against transformation vector sequence

T-DNA	Transgenic event	Number of reads		Average length	Average insert size
		Mapped reads	Paired mapped reads		
pSRT-Bt9	Illmi^a^	8125	648	121	468
	5	11,539	2790	122	479
	30	8371	1792	122	469
	109	9767	2336	121	535

^a^Control parent cultivar

**Fig. 2:**
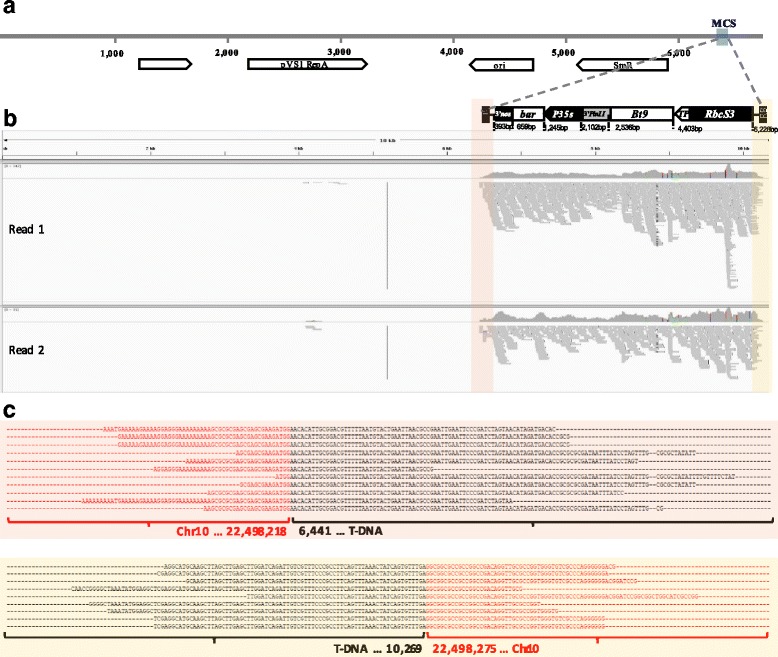
Molecular characterization of transgenic rice using NGS read alignments. **a** Illustration of transformation plasmid pPZP200 containing T-DNA used for *Agrobacterium*-mediated transformation to create SNU-Bt9–5, SNU-Bt9–30, and SNU-Bt9–109. MCS, multiple cloning site. **b** Detailed example of IGV results. Horizontal lines on the sequence track (top of the panel) indicate the reference sequence (i.e., T-DNA inserted transformation plasmid vector sequence). Featured tracks exhibit a paired orientation (upper panel = read 1, lower panel = read 2). Colored boxes indicate junction region containing reads spanning both the T-DNA border and the genomic flanking sequence. **c** Sequence alignments of junction-spanning reads (upper = left border flanking sequences, lower = right border flanking sequences). Red and black nucleotides indicate rice chromosome and T-DNA, respectively

**Fig. 3:**
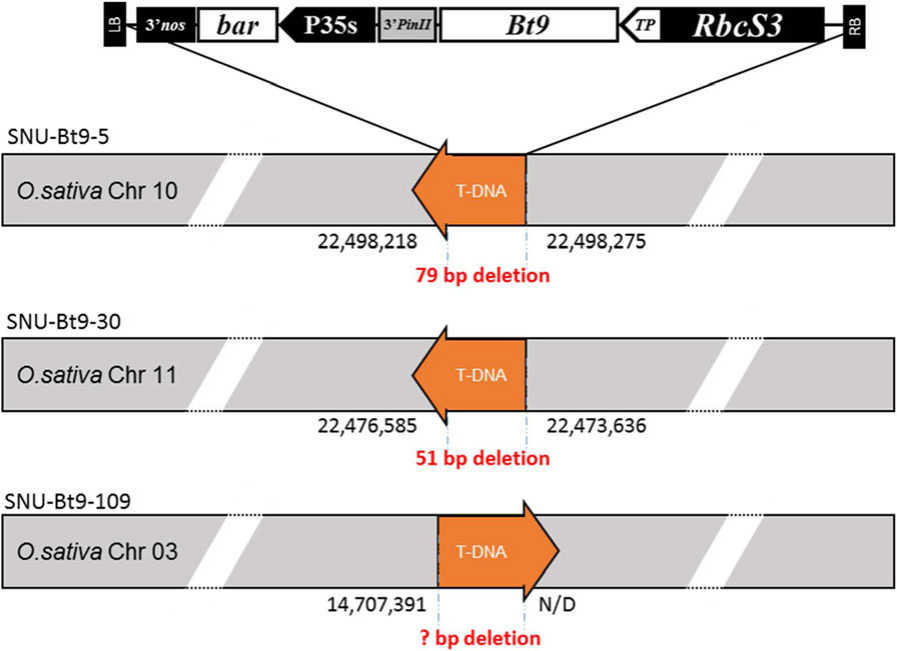
Representation of deduced loci of a T- DNA insertion in a rice chromosome

Reads that partially aligned with both ends of the transgene border region were collected (Fig. 2a and b) based on their mapping position. Then, selected reads were aligned to the entire T-DNA sequence to identify the flanking site. The results represented insert junctions on rice chromosomes (Fig. 2c). Reads spanning junction regions between the host genome and transgene obtained from the SNU-Bt9–5 rice mapped perfectly to rice chromosome 10 from 22,498,218 to 22,498,279 bp with 79-bp deletions. The SNU-Bt9–30 rice event was properly mapped to rice chromosome 11 from 22,473,585 to 22,473,636 bp with 51-bp deletions (Table 3 and Fig. 3). Both transgenic events successfully detected a single copy and a single locus within the rice genome, and both results were identical to those obtained by the Southern blot-based detection method [21].

**Table 3:** Inserted T-DNA locus information of GM rice events

T-DNA	Event	Mapped chromosome					Insert orientation
		Chr.	Accession	Start	End	Deletion	
pSRT-Bt9	5	10	AP014966.1	22,498,218	22,498,297	79	3′-5’
	30	11	AP014967.1	22,473,585	22,473,636	51	3′-5’
	109	3	AP014959.1	N/D	14,707,459–14,707,391	-	N/D

N/D, not detectable

Although integration sites of SNU-Bt9–109 rice were not identified using the method described here (Table 3 and Fig. 3), the integration site near the right border (RB) was found on chromosome 3 from 14,707,459 to 14,707,391 bp. Flanking sequences near the left border (LB) region were not identified. BLASTN analysis (using the NCBI nr database) showed that the junction between the LB region and the host genome showed high similarity to the “Gene trapping Ds/T-DNA vector pDsG8 (e-value: 4e-28)” and the *Solanum tuberosum* proteinase inhibitor gene (e-value: 6e-28). However, the *S. tuberosum* gene was regarded as an artifact due to its short query and low specificity.

To validate the above results, we designed primers based on the obtained junction sequence reads (Additional file 1: Table S1). Our PCR results verified that insertion detection of the two transgenic rice events was successfully characterized using NGS. Moreover, the junction sequence of SNU-Bt-109 was also detected by flanking PCR using nearby LB sequences (Additional file 1: Figure S2).

### Determining T-DNA rearrangement

To determine the T-DNA sequence, we calculated insert size distributions using reads of mapped pairs against the transgenic plasmid DNA (Additional file 1: Figure S3). By calculating insert size, it is possible to decide whether the inserted DNA has been rearranged. Average insert sizes were 479, 469, and 535 bp for SNU-Bt9–5, SNU-Bt9–30, and SNU-Bt9–109, respectively, which properly matched with the sizes prepared in library construction (Additional file 1: Figure S4). It assumed that there were no internal rearrangements or duplications inside the T-DNA. The results correspond to those of whole T-DNA retrieval by genomic DNA PCR and sequencing analysis in our previous paper [21].

### Possible presence of backbone sequences in transgenic plants

Unintended genomic changes may occur during the development of new GM plants. It is possible for plasmid backbone sequences to be integrated into a host’s genome during *Agrobacterium*-mediated transformation [10]. Therefore, sequence alignments were visualized with IGV to detect possible contamination of plasmid backbones. No reads were mapped to the plasmid backbone structure (Additional file 1: Figure S5 and S6). This finding demonstrates that backbone-derived sequences were not introduced into these transgenic genomes.

## Discussion

The purpose of this study was to develop a simple, fast, and accurate method for the molecular characterization of transgenic plant genomes for researchers who have limited resources for bioinformatic analyses. Our transgene detection method is intended for use when the DNA sequence of the transformation vector and the reference genome sequence are available. However, genomic reference sequences and a priori information of transgenes are not always available, which represents a limitation for the application of this method that must be further investigated.

A sufficient amount of high-quality raw data is a prerequisite for this whole genome re-sequencing approach for event characterization. In this study, we achieved high coverage (average sequencing depth, 68×) across the entire genome of *O. sativa* (389 Mb), and we consequently obtained a sufficient amount of junction-spanning reads that allowed us to obtain reliable and robust results. The quantity of sequencing coverage varies considerably (from 10× to over 75×) depending on analytical methods [12, 17–19, 25, 26]. Although the question of what constitutes sufficient coverage for detecting the presence of inserts is debatable, many publications have suggested that the higher the sequencing depth is, the better it is for achieving precise molecular characterization of GM events [27, 28]. However, higher coverage is associated with higher cost; therefore, a suggestion for standardizing the appropriate amount of NGS data for molecular characterization of GM events is needed [19, 29]. Recently, in an alternative approach, Zastrow-Hayes et al. [12] developed the SbS method, which was designed to enrich the target sequence where the adequate depth for SbS is greater than 100×. Guttikonda et al. [19] demonstrated that the SbS method coupled with NGS can answer the above-mentioned concerns pertaining to achieving precise molecular characterization of GM crops.

For obtaining a precise insert junction location using NGS, a read alignment algorithm is used, and this strategy has a strong influence on computational running time. We compared two published reference-based methods for the molecular characterization of GM plants [17, 18]. To detect junction sequences from NGS reads, Kovalic et al. [17] searched on the basis of sequence similarity to the appropriate query sequences using the local alignment software BlastAll (version 2.2.21). The host genome of the native soybean and transformation vector were used as queries for the selection of reads. However, Blast alignment is computationally costly for aligning whole-genome high-throughput sequence reads and requires large amounts of memory. Another study mapped to the sequence of the host reference genome and the plasmid of GM rice events using the short read aligner BWA with a single-end strategy [18]. We tested several combinations of alignment software, such as Blast, Bowtie, BWA, and BWA-MEM (data not shown). In this study, BWA-MEM, which provides end-to-end and local or chimeric alignments, offered the best performance as it was the most accurate method and required a relatively short computation time. BWA-MEM may have longer runtimes than Bowtie or the BWA-aln algorithm, but it produces more accurate and reliable results.

Another consideration is that the integration of T-DNA occurs within repetitive regions of host genomes [19]. Plant repetitive sequences pose some challenges for detecting a junction locus during the mapping step. Illumina sequencers produce ‘short’ reads due to the nature of the chemistry, which may cause an increase in the percentage of ambiguously or incorrectly mapped reads. This limitation could be overcome by using the Pacific BioSciences sequencing platform (PacBio), which can generate long-read sequencing data [25]. Longer reads are very promising for sequence alignment even though they require a well-annotated host genome.

Although this new methodology provides detailed information for the molecular characteristics of SNU-Bt9–5 and SNU-Bt9–30, the precise junction location of SNU-Bt9–109 was not identified. This is due to the challenging nature of current sequencing and bioinformatic technologies (e.g., short read length of Illumina reads) that have been already identified in several studies [19]. Therefore, it could be argued that this mapping strategy alone is insufficient to assess the applicability of potential methods for molecular characterization of transgenic events. In addition, we suggest that complete molecular characterization of GMOs using NGS and bioinformatics should be coupled with experimental methods (i.e., primer walking or further Sanger sequencing).

## Conclusion

Previous studies have demonstrated that NGS can facilitate the molecular characterization of GMOs in light of its high throughput, continuously decreasing costs and the development of a diverse range of bioinformatics tools. However, NGS does not currently provide a standardized workflow model outlining the necessary steps for the molecular characterization of GM crops. One major reason for this is that NGS data analysis requires a profound understanding of bioinformatics, as the NGS methods produce vast amounts of data demanding special knowledge of computer science. In this regard, the novelty of our study is that it empirically tests GM rice that is currently under development by biologists in a general research laboratory, not experts in bioinformatics or computer science or a multinational seed company. In this study, we successfully detected the size and copy number of inserts, location information of flanking regions, and the absence of plasmid vector sequences with a simple and quick screening method. Although the method described here focused on identifying T-DNA insertions into the rice genome by whole genome re-sequencing, NGS can also be applied to de novo approaches and full assemblies of T-DNA.

## Additional files

Additional file 1: Table S1. Primer pairs used in PCR. **Figure S1.** Circular map of T-DNA construct. **Figure S2.** PCR confirmation of the junction site in three transgenic rices. **Figure S3.** Insert size distribution of three transgenic rice plants. **Figure S4.** Electropherogram of the fragment size of sequencing library using Caliper GX. **Figure S5.** View of alignments mapped against T-DNA (6.2 kb) using IGV. **Figure S6.** View of alignments mapped against whole transformation plasmid (~1.3 kb) using IGB. (PDF 1285 kb)

Additional file 2: Source code for T-DNA insert site analysis. (TXT 890 bytes)

## Abbreviations

2,4-D: 2,4-dichlorophenoxyacetic acid
BWA-MEM: Burrows-Wheeler Aligner software with maximal exact matches
GM: Genetically modified
GMO: Genetically modified organism
IGV: Integrative Genomic Viewer
LB: Left border
NGS: Next-generation sequencing
RB: Right border
rbcS3: Ribulose-1,5-bisphosphate carboxylase/oxygenase small subunit 3
SbS: Southern-by-sequencing
T-DNA: Transfer DNA
