# Supplementary material for: A bioinformatics approach for identifying transgene insertion sites using whole genome sequencing data
Source: BMC Biotechnol. 2017 Aug 15;17:67. doi: 10.1186/s12896-017-0386-x (PMC5558722; doi:10.1186/s12896-017-0386-x)
Supplement: Supplementary file 1 — Primer pairs used in PCR. Figure S1. Circular map of T-DNA construct. Figure S2. PCR confirmation of the junction site in three transgenic rices. Figure S3. Insert size distribution of three transgenic rice plants. Figure S4. Electropherogram of the fragment size of sequencing library using Caliper GX. Figure S5. View of alignments mapped against T-DNA (6.2 kb) using IGV. Figure S6. View of alignments mapped against whole transformation plasmid (~1.3 kb) using IGB. (PDF 1285 kb) [file 12896_2017_386_MOESM1_ESM.pdf]

Additional File 1: Detailed methods for experimental validation and Figure S1 to S6.

Methods in detail:

Polymerase Chain Reaction (PCR)

PCR were performed using flanking region sequences. PCR amplifications were performed with the primers listed in Table S1 and S2 using i-StarTaq DNA polymerase (Cat No. 25161, Intron Biotechnology Co., Ltd.). All PCRs were conducted with the following cycling profile: 5 min at 94 °C, 35 cycles of (30 sec at 94 °C, 30 sec at 60 °C, 40 sec at 72 °C), followed by 5 min at 72 °C. The expected sizes; RB of Bt9-5, 812 bp; LB of Bt9-5, 700 bp; RB of Bt9-30, 731 bp, LB of Bt9-30, 568 bp; RB of Bt9-109, bp; LB of Bt9-109 bp. β-tubulin was used as control gene (expected size 160 bp). Primers used in PCR were listed in Table S1. PCR products were separated by 1% agarose gel electrophoresis.

Table S1. Primer pairs used in PCR

Sample Name	RB	LB
Bt9-5_gDNA_F	GAGGCAGTACCACCAGTAGC	GCCGGTCTTGCGATGATTAT
Bt9-5_gDNA_R	GCGCACGGATATACCATTGT	TCTCTTCGCCTCTCGTCAAC
Bt9-30_gDNA_F	CCACTTAGCGGATCTCATTG	GCCGGTCTTGCGATGATTAT
Bt9-30_gDNA_R	GCGCACGGATATACCATTGT	ACCTCTCTGCGTAATCTCCAATTT
Bt9-109_gDNA_F	GCCTCTTGTTCATTGCAGTC	GCCGGTCTTGCGATGATTAT
Bt9-109_gDNA_R	GCGCACGGATATACCATTGT	GCCACTGATGTGGTGCTGTC
β-tubulin_F	CTACCTCACGGCATCTGCTATGT	
β-tubulin_R	GTCACACACACTCGACTTCACG	

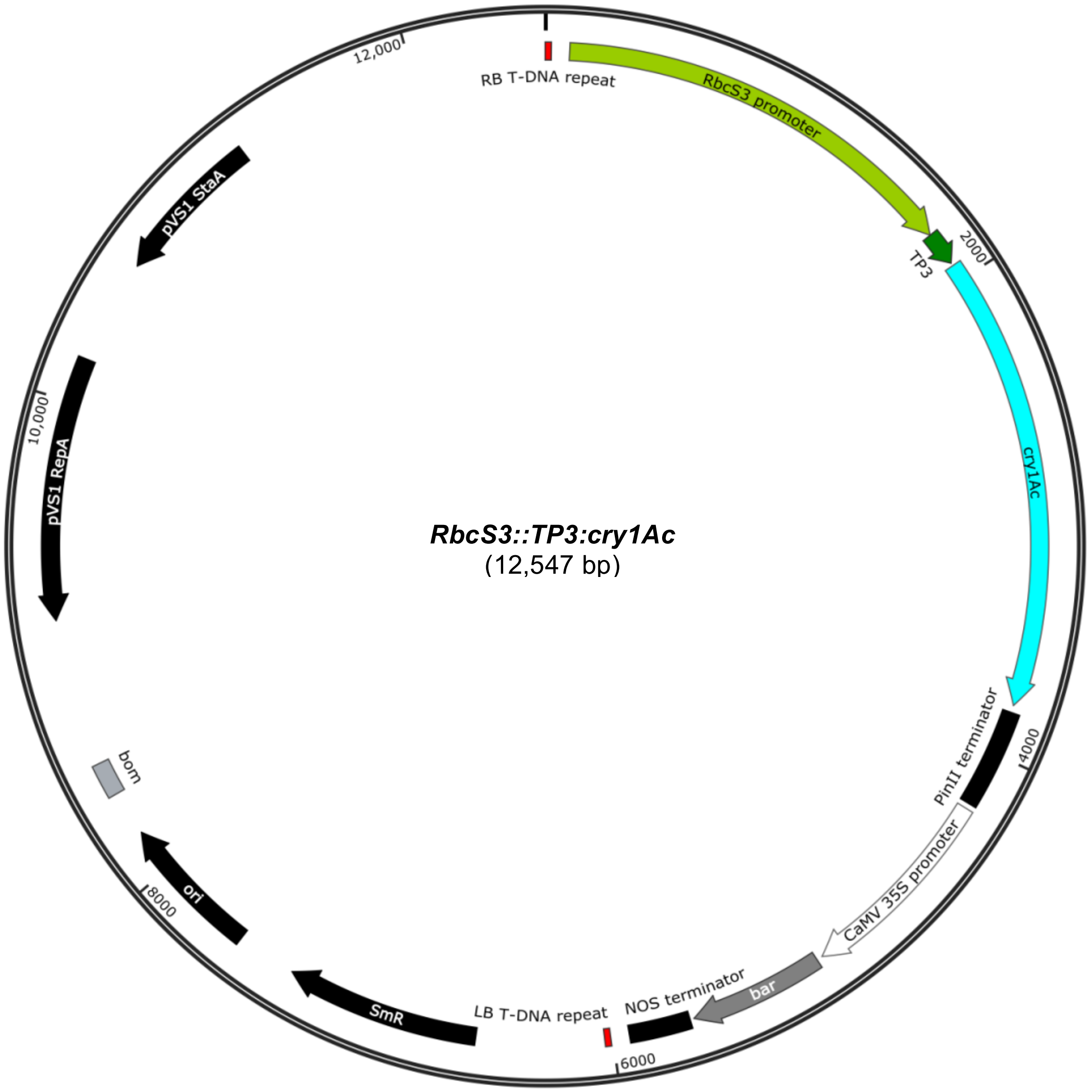

Figure S1. Circular map of T-DNA construct.

The vector construct *RbcS3:TP3:cry1Ac* consists with *cry1Ac* gene controlled under *RbcS3* promoter and a herbicide resistant selection marker, bar gene under the *CaMV 35S* promoter between left (L) and right (R) T-DNA borders. The plasmid DNA backbone comes from pPZP200 including replication (*pVS1 RepA*) and stability (*pVS1 StaA*) region in *Agrobacterium*. *RbcS3*, Rubisco small subunit 3; *TP3*, transit peptide 3; *PinII*, potato proteinase inhibitor II; *NOS*, nopaline synthase terminator; *SmR*, spectinomycin resistance gene.

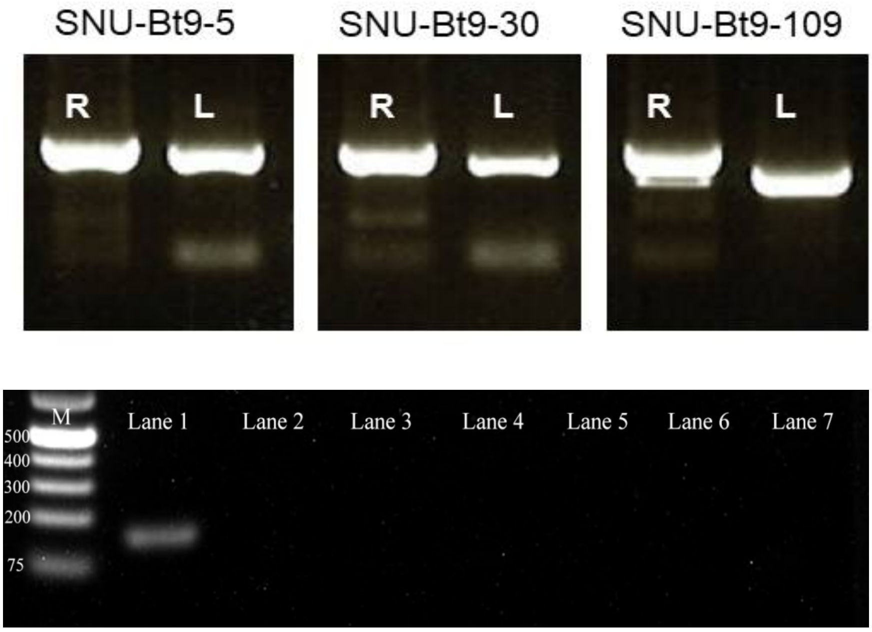

Figure S2. PCR confirmation of the junction site in three transgenic rices (upper). “R” and “L” represent near right border and left border of T-DNA region, respectively. PCR primers were designed based on the junction sequence reads detecting from bioinformatics analysis. Primers were also tested for gDNA of Illmi (bottom). M, 1Kb ladder (bp); Lane 1 is β-tubulin as positive control; Lane 2-6, PCR product of Bt9-5_gDNA_F, Bt9-5_gDNA_R, Bt9-30_gDNA_F, Bt9-30_gDNA_R, Bt9-109_gDNA_F, and Bt9-109_gDNA_R, respectively.

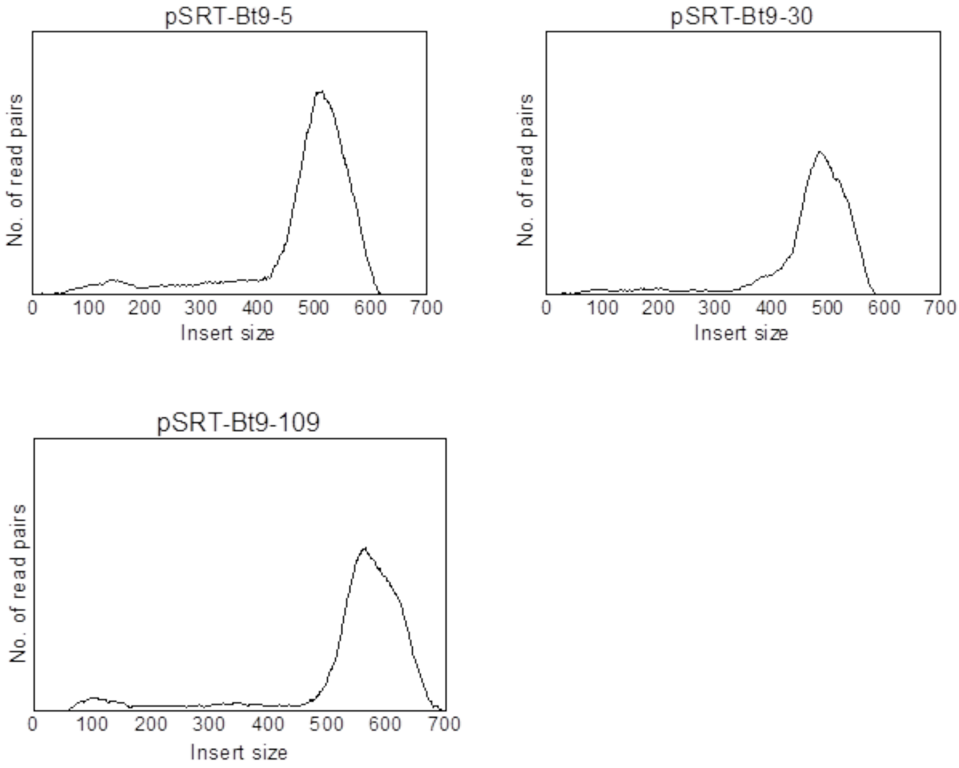

Figure S3. Insert size distribution of three transgenic rice plants. The graph represents average distances of paired-end reads aligned against the whole transformation plasmid. Average insert sizes were 479, 469, and 539 bp for SNU-Bt9-5, SNU-Bt9-30, and SNU-Bt9-109, respectively. The observation of an average distance of paired mapped reads was consistent with the sequencing library sizes.

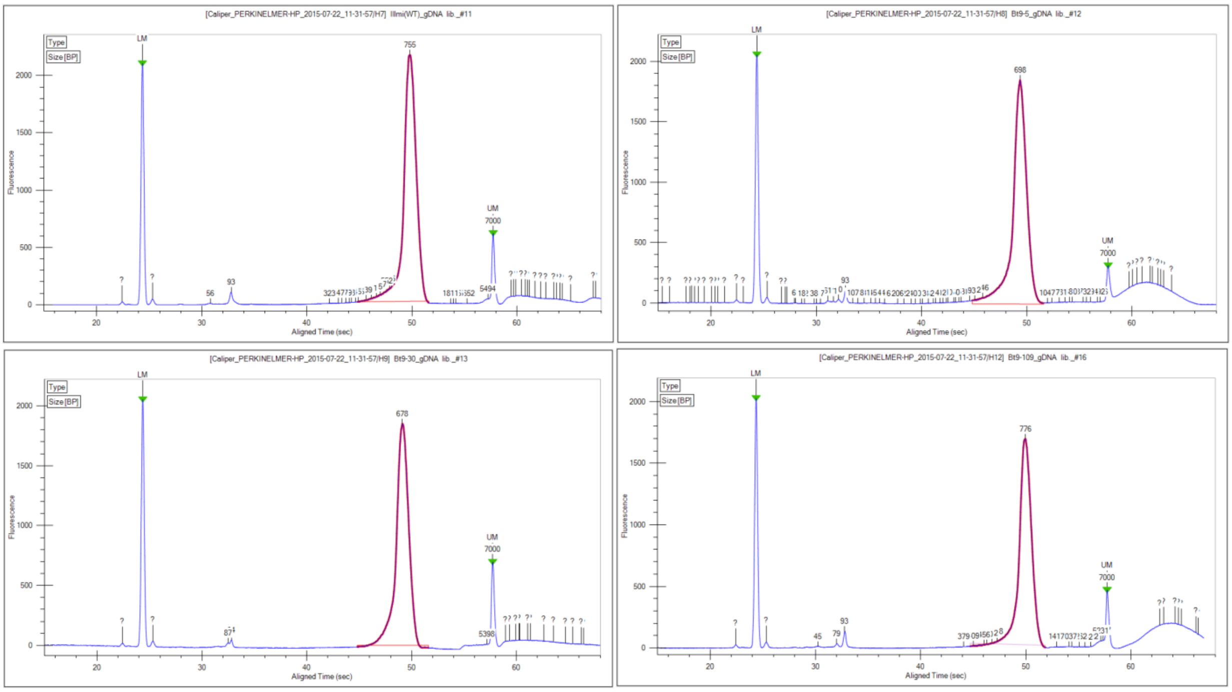

Figure S4. Electropherogram of the fragment size of sequencing library using Caliper GX machine. The average sizes of constructed library were 755 bp, 698 bp, 678 bp, 776 bp of Illmi, SNU-Bt9-5, SNU-Bt9-30, and SNU-Bt9-109, respectively.

SNU-Bt9-5

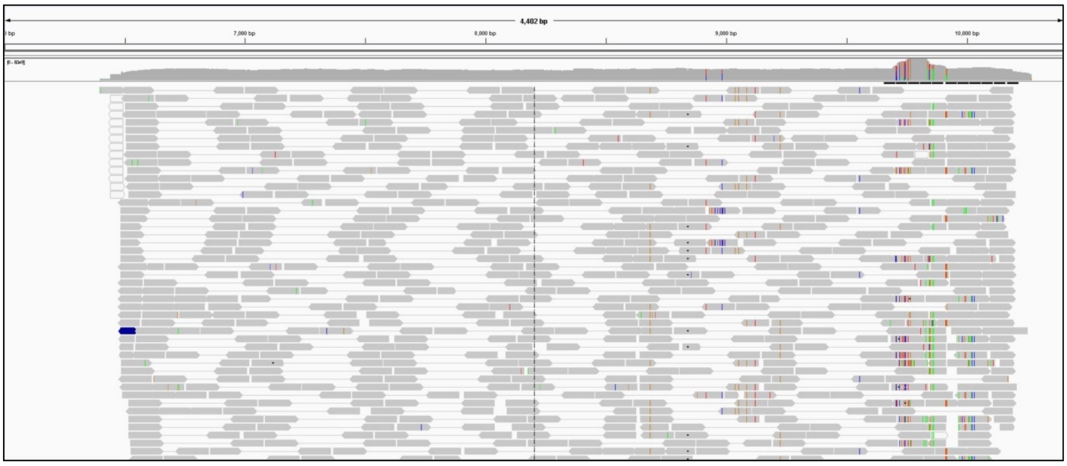

SNU-Bt9-30

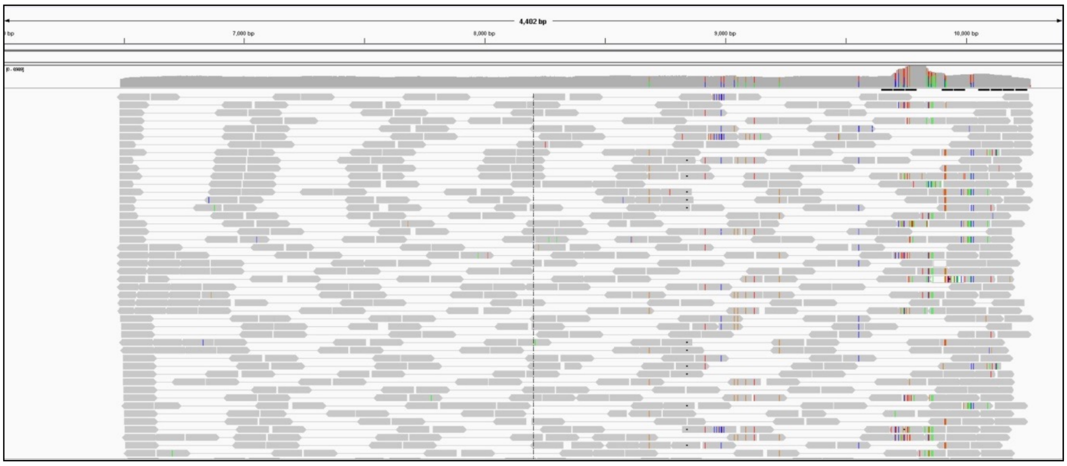

SNU-Bt9-109

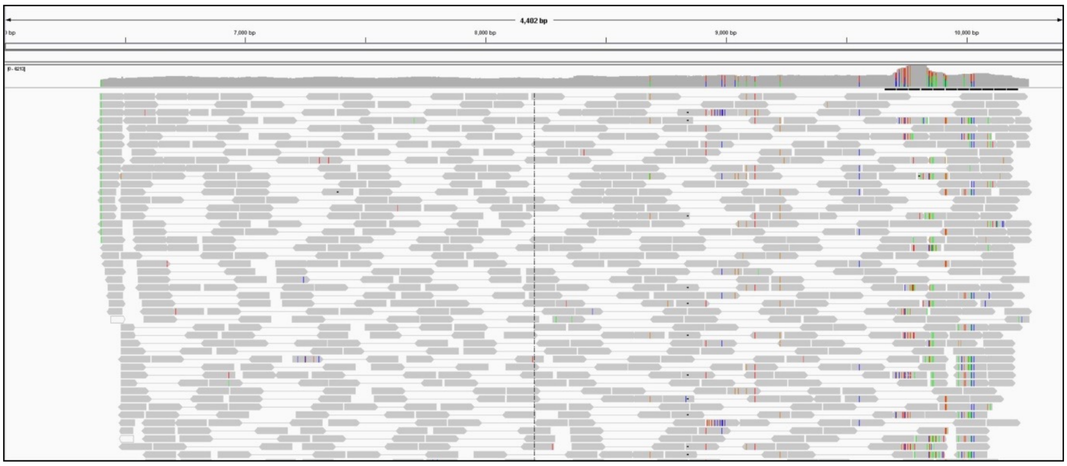

Figure S5. View of alignments mapped against T-DNA (6.2 kb) using IGV. Reads were obtained from Illumina (San Diego, CA, USA) Hiseq2500 platform and aligned with bwa-mem (http://bio-bwa.sourceforge.net/). Alignment reads are represented as gray polygons by paired orientation and mismatched position relative to the reference were colored.

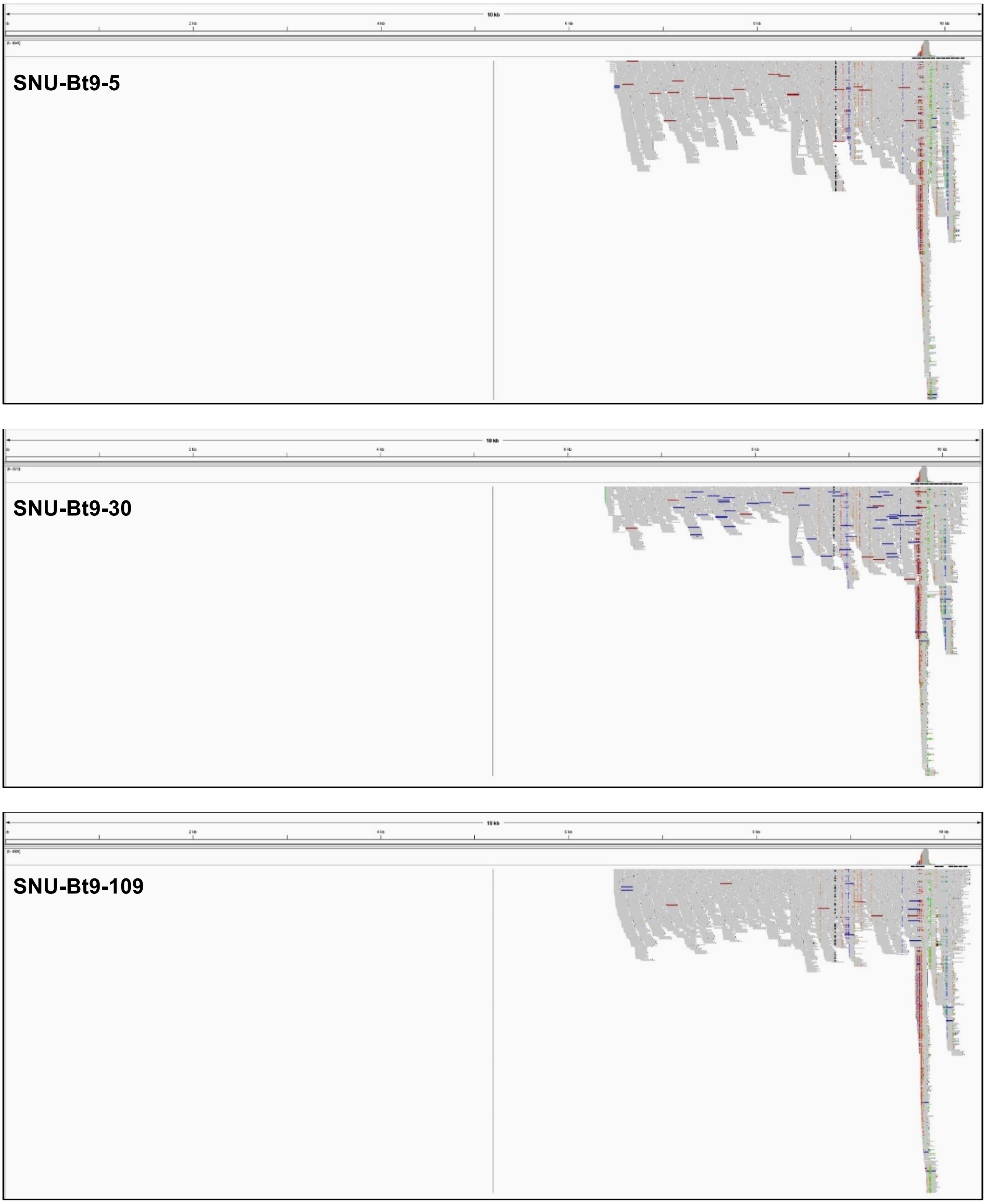

Figure S6. View of alignments mapped against whole transformation plasmid (~1.3 kb) using IGV. Vector backbone pPZP200 containing T-DNA sequences were used to detect backbone contamination. There is no detectable backbone DNA fragment among the three transgenic rice plants.
